# The clinical and genomic distinctions of Class1/2/3 BRAF-mutant colorectal cancer and differential prognoses

**DOI:** 10.1186/s40364-022-00443-8

**Published:** 2023-01-25

**Authors:** Yungchang Chen, Hao Sun, Yanhong Deng, Yutong Ma, He Huang, Yang Liu, Yaru Zhang, Hongyu Zhang, Sheng Ye, Mingyan E, Hongqiang Guo, Mengmeng Wu, Chunman Wu, Xingxiang Pu, Xinggui Chen, Chaoyong Liang, Qiuxiang Ou, Huawei Weng, Xue Wu, Yang Shao, Anxin Gu, Tongyu Lin

**Affiliations:** 1 Department of Medical Oncology, Sichuan Cancer Center, School of Medicine, Sichuan Cancer Hospital and Institute, University of Electronic Science and Technology of China, No. 55, Section 4, South Renmin Road, Sichuan 610041 Chengdu, China; 2 Department of Gastrointestinal Cancer Center, Chongqing University Cancer Hospital, 400030 Chongqing, China; 3 Department of Medical Oncology, The Sixth Affiliated Hospital of Sun Yat-sen University, 510655 Guangzhou, China; 4 Geneseeq Research Institute, Nanjing Geneseeq Technology Inc, 210000 Nanjing, China; 5 Department of Medical Oncology, State Key Laboratory of Oncology in Southern China, Collaborative Innovation Center of Cancer Medicine, Sun Yat-sen University Cancer Center, 510060 Guangzhou, China; 6 Department of Pathology, Sichuan Cancer Center, School of Medicine, Sichuan Cancer Hospital and Institute, University of Electronic Science and Technology of China, 610041 Chengdu, China; 7 Department of Medical Oncology, The Fifth Affiliated Hospital of Sun Yat-sen University, 519000 Zhuhai, China; 8 Department of Medical Oncology, The First Affiliated Hospital of Sun Yat-sen University, 510080 Guangzhou, China; 9 Department of Radiation Oncology, Harbin Medical University Cancer Hospital, 150 Haping Road, Nangang District, 150040 Harbin, Heilongjiang China; 10 Department of Medical Oncology, The Affiliated Cancer Hospital of Zhengzhou University, Henan Cancer Hospital, 450008 Zhengzhou, China; 11 Department of Thoracic Medical Oncology, Hunan Cancer Hospital and The Affiliated Cancer Hospital of Xiangya School of Medicine, Central South University, 283 Tongzipo Road, Yuelu District, 410013 Changsha, China; 12 Department of Medical Oncology, Cancer Center, Affiliated Hospital of Guangdong Medical University, 524023 Zhanjiang, China; 13 Department of Medical Oncology, Guangxi Medical University Cancer Hospital, 530021 Nanning, China; 14 School of Public Health, Nanjing Medical University, 211166 Nanjing, China

**Keywords:** *BRAF*, Colorectal cancer, Next-generation sequencing, Prognosis

## Abstract

*BRAF* mutations are the oncogenic drivers in colorectal cancer and V600 mutations (Class1), which lead to RAS-independent active monomers, are the most common mutation types. *BRAF* non-V600 mutants can be further classified as RAS-independent active dimers (Class2) and RAS-dependent impaired kinase (Class3). We retrospectively reviewed the mutational profiles of 328 treatment-naïve colorectal tumors with *BRAF* mutations detected using capture-based hybrid next-generation sequencing targeting 400 + cancer-related genes. The clinical and genetic distinctions of patients harboring Class1/2/3 *BRAF* mutations were investigated, which revealed that tumors with Class1 *BRAF* mutations showed more unique genomic profiles than those with Class2/3 mutations. Also, by using an external dataset from cBioPortal, we demonstrated that patients with Class3 *BRAF* mutations had the best survival outcomes compared to the other two subgroups. These findings promoted the development of anti-BRAF strategies by distinguishing *BRAF* mutant subgroups.

**Supplementary Information:**

The online version contains supplementary material available at 10.1186/s40364-022-00443-8.

***To the editor,***

*BRAF* mutation is considered to be an oncogenic driver in colorectal cancer (CRC) and V600E is the most dominant *BRAF* mutation [1]. Mutations occurring at V600 of *BRAF* such as V600E/K/D/R/M cause the expression of RAS-independent active monomeric proteins, which are grouped as Class1 *BRAF* mutations. Other non-V600 *BRAF* mutations can be classified based on signaling mechanism and kinase activity, including RAS-independent active dimers (Class2) and RAS-dependent impaired kinase (Class3) [2, 3]. The molecular features of the three classes of *BRAF* mutations varied and could lead to different treatment strategies [4]. For instance, canonical BRAF inhibitors, such as encorafenib and vemurafenib, directly target the monomeric BRAF protein, to which only the patients carrying Class1 *BRAF* mutations were sensitive [5]. Thus, comprehensively studying the distinctions in signaling transduction and other genetic features among patients carrying different classes of *BRAF* mutations may inspire the combination strategies of existing BRAF inhibitors and other targeted therapies. Also, the development of next-generation BRAF inhibitors targeting the dimeric BRAF protein is urgently needed.

In this multicenter retrospective study, the treatment-naïve tumor samples with *BRAF* mutations collected from 328 CRC patients were analyzed using next-generation sequencing (NGS, Fig.S1A; Class1: *N* = 246, Class2: *N* = 29, Class3: *N* = 53). The incidence of Class1 mutation in this cohort (75%, 246/328) was comparable to previous studies (79%, 92/117) [2]. All Class1 *BRAF* mutations were V600E, while Class2 and Class3 mutations were predominantly G469 (53%) and D594 (56%), respectively (Fig. 1A). No differences in patient’s age, sex, and stage were observed among the three subgroups, but the anatomical location of the tumor differed among subgroups, particularly between Class1 and Class3 (*p* = 0.027, Table S1).

**Fig. 1:**
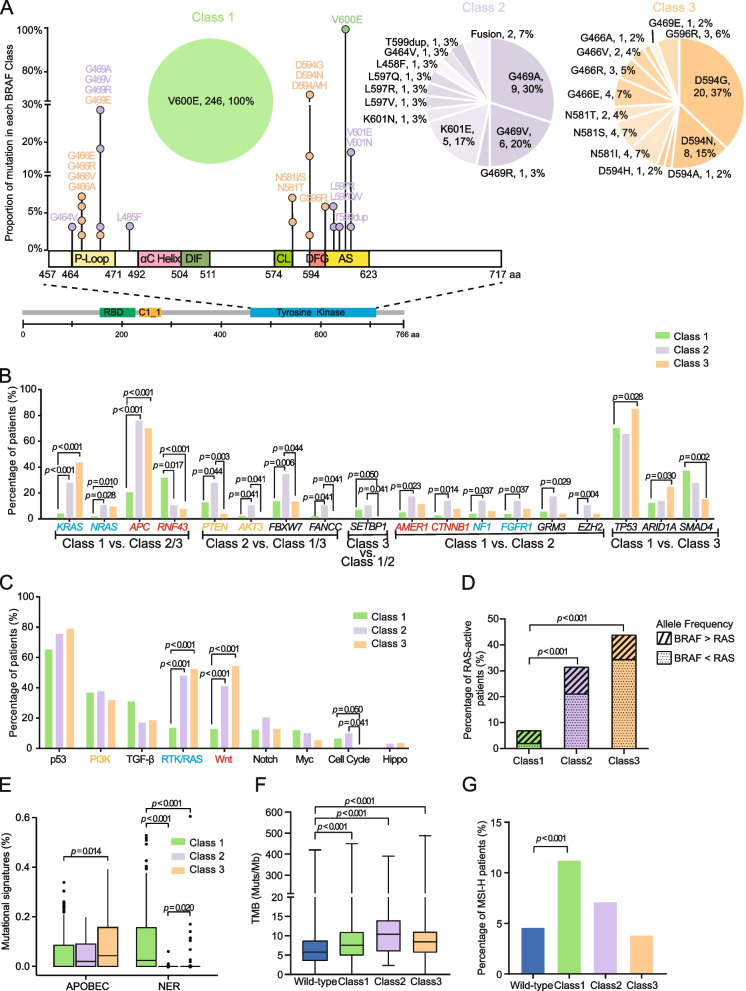
The genomic features of tumors with Class1 *BRAF* mutations are more unique than those with Class2/3 mutations. **A** The positions of detected mutations in each class (Class1: green; Class2: purple; Class3: orange) along the tyrosine kinase domain of *BRAF* gene are illustrated by the lollipop chart, whose frequencies are shown by the pie charts. **B** The proportion of patients with genomic alterations across three *BRAF* classes. All genes whose mutational frequencies were over 10% in either Class1 or 2 or 3 subgroup were included for analysis and significant genes (*p* < 0.05 based on Fisher’s exact test) are grouped based on enrichment patterns indicated below the gene names (e.g., Class1 vs. Class2/3 subgroup includes genes whose mutational frequencies were significantly different in Class1 compared to both Class2 and Class3). Genes involved in PI3K, RTK/RAS, and Wnt signaling pathway are colored in orange, blue, and red, respectively, and the rest significant genes are black. **C** Frequencies of pathway alterations by *BRAF* classes. **D** Frequencies of patients harboring *KRAS/NRAS/HRAS* activation mutations are shown in each *BRAF* class. The allele frequency differences between RAS-active mutation and *BRAF* mutation are labeled by slash (BRAF > RAS) and dots (BRAF < RAS), respectively. **E** Mutational signature analysis was performed based on the COSMIC database. APOBEC (apolipoprotein B mRNA editing catalytic polypeptide-like enzyme) is associated with Signature 2 and Signature 13, and NER (nucleotide excision repair) is related to Signature 22. Statistical analyses are performed between all pairs of subgroups using the Mann-Whitney test and the significant *p* (< 0.05) values are labeled. **F** The levels of TMB in *BRAF-*wt and three *BRAF* classes are shown by the boxplot and the significant *p* (< 0.05) values based on the Mann-Whitney test are labeled. **G** Percentage of MSI-H patients by *BRAF* status and Fisher’s exact tests are performed in all pairs of comparison. Abbreviation: RBD: receptor-binding domain; DIF: dimerization interface; CL: catalytic loop; DFG: Asp-Phe-Gly motif; AS: activation segment

Somatic mutation profiling revealed that the most frequently mutated gene in Class1 and Class3 was *TP53* (66.9% and 84.9%) while that in Class2 was *APC* (75.9%, Fig.S1B). *KRAS*, *NRAS*, and *APC* mutations were significantly enriched in the Class2/3 subgroups, while the mutational frequency of *RNF43* was significantly higher in the Class1 subgroup. The genes involved in PI3K signaling pathway, including *PTEN* and *AKT3*, were significantly more frequently mutated in Class2 (Fig. 1B). In addition, Wnt or RTK/RAS signaling pathway alterations were significantly more common in Class2/3 subgroups compared to Class1, but pathogenic cell cycle pathway alterations were only detected in Class1/2 (Fig. 1C). Then, we compared the allele frequencies (AFs) of *BRAF* mutations and concurrent *KRAS/NRAS/HRAS* mutations that were reported to cause RAS signaling activation. As shown in Fig. 1D, the proportions of patients harboring RAS activating mutations in Class2 (31%) and Class3 (43%) were significantly higher than that in Class1 (7%, *p <* 0.001). In Class2/3, the AFs of *BRAF* mutations were commonly lower than the concurrent RAS activating mutations, indicating that these *BRAF* mutations might be subclonal. Furthermore, the mutational signature associated with nucleotide excision repair (NER) was significantly enriched in tumors with Class1 *BRAF* mutations, but the mutational signature related to the apolipoprotein B mRNA editing catalytic polypeptide-like (APOBEC) enzyme was more commonly identified in Class3 compared to Class1 (Fig. 1E). The tumor mutational burden (TMB) of patients harboring *BRAF* mutations was significantly higher than that of patients with wildtype *BRAF* (median: 5.8), while Class2 *BRAF*-mutant tumors demonstrated non-significantly higher TMB than the other two subgroups (median: 10.4 vs. 7.6 and 8.4, Fig. 1F). However, microsatellite instability-high (MSI-H) tumors were slightly more common in Class1 compared to Class2/3 (Class1: 11% vs. Class2: 7% vs. Class3: 4%, Fig. 1G), but significantly when compared with wildtype subgroup (5%, *p <* 0.001).

Due to the lack of survival data in our cohort, we utilized an external dataset from cBioPortal which contained 455 unresectable metastatic CRC patients to investigate the association between prognosis and *BRAF* mutation subtypes (wildtype: *N* = 396, Class1: *N* = 38, Class2: *N* = 8, Class3: *N* = 8, Others: *N* = 5) [6]. First, similar analyses on genetic characteristics including concurrent RAS-activating mutations, MSI-H, and TMB levels were performed to validate the findings observed in our cohort. As shown in Fig. 2A, Class1 *BRAF* V600E mutation was not concurrent with any RAS activating mutations, which were detected in 62% of patients with wildtype *BRAF* and 50% of those in Class3 (*p <* 0.001). Similar to our cohort, the proportion of MSI-H patients was the highest in Class1 (24%, Fig. 2B). Even though no significant difference in TMB was observed among those subgroups in this external cohort, a trend of higher TMB in *BRAF-*mutated tumors than *BRAF-*wildtype tumors was observed (Fig. 2C), which also supported the genetic similarity between the two cohorts. Then, we investigated the survival outcomes of patients with Class1/2/3 *BRAF* mutations. As shown in Fig. 2D-E, patients with Class1 *BRAF* mutants had the worst progression-free survival (PFS; median: 5.0 months) and overall survival (OS; median: 12.7 months) whereas patients in Class3 demonstrated the best prognosis (median PFS: 7.3 months; median OS: 31.9 months). Considering the restricted cohort size for patients with Class2/3 *BRAF* mutations, we exploited a second independent dataset for survival analysis [7]. A similar trend of better OS was observed in patients with Class 3 *BRAF* mutations versus the other two subtypes (Fig. S2), although the sample size of this cohort was still limited (Class1: *N* = 27, Class2: *N* = 5, Class3: *N* = 8) due to the low frequency of Class2/3 *BRAF* mutations.

**Fig. 2:**
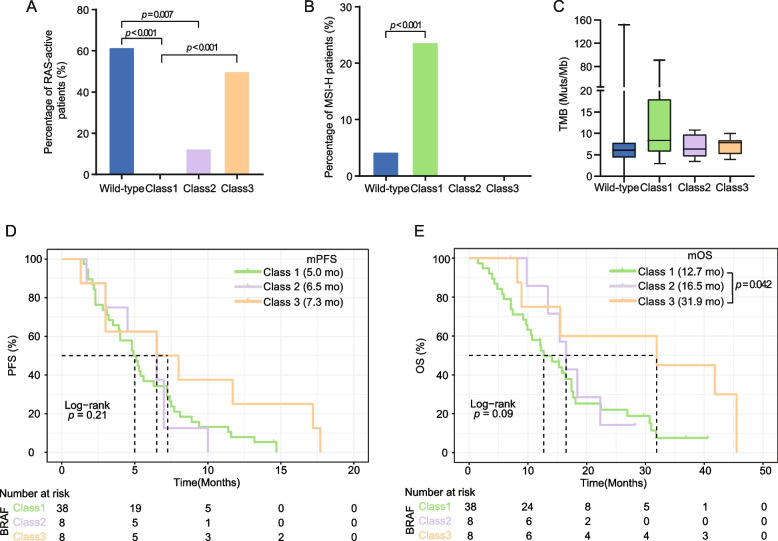
Patients harboring Class 3 *BRAF* mutations show better prognosis than those with Class 1/2 mutations analyzed in a public cBioPortal cohort. Percentages of patients with (**A**) RAS-active alterations and (**B**) MSI-H are shown by *BRAF* subgroups. (**C**) The distribution of TMB. (**D**-**E**) Kaplan-Meier analyses of progression-free survival and overall survival for patients with different classes of *BRAF* mutations

Matsumoto et al. [8] reported a strong association between *RNF43* mutations and *BRAF* V600E, which was consistent with our observation (Fig. 1B). However, other genes in the Wnt signaling pathways, e.g., *APC*, *AMER1*, and *CTNNB1* showed the opposite trend and the percentage of patients with alterations in Wnt pathway were significantly higher in Class 2/3 than Class1 (Fig. 1C). Notably, the TMB levels of each *BRAF* subgroup in our cohort were not in accordance with the public cBioPortal cohort [6], which might be due to the restricted cohort size and the different targeted NGS panels used for TMB estimation. In addition, the large difference in cohort size between Class1 and Class2/3 both in our cohort and the external dataset should be noted, implying the warrant of further validation with a larger sample size of Class2/3 *BRAF* mutations. Also, the zygosity status (homozygous vs. heterozygous) of the *BRAF* mutations was not evaluated in the current NGS pipeline and the external dataset, which was reported to be associated with the response to BRAF inhibitor treatment [9]. Thus, to comprehensively interpret treatment sensitivity and prognosis between different zygosity status, further studies are needed. The prognosis of three *BRAF* subgroups analyzed here was supported by a previous study that reported the longest PFS and OS in patients with Class3 *BRAF* mutations [2]. As the available BRAF inhibitors only target *BRAF* V600 mutations, patients with Class2/3 *BRAF* mutations are not sensitive to them [10]. Encouragingly, a second-generation BRAF inhibitor, BGB-3245, targeting both monomer and dimer forms of active BRAF proteins is in the early phase of clinical trial (NCT04249843), which might expand the benefits to patients with non-V600 *BRAF* mutations. In addition, due to the high dependency on EGFR signaling, Class3 *BRAF* mutations were proven to respond to anti-EGFR therapy, indicating a bright future of combination therapies in patients with non-V600 *BRAF* mutations [4].

In conclusion, the CRC tumors harboring Class1 *BRAF* mutations demonstrated more unique genetic profiles than those with Class2/3 mutations and patients with Class3 *BRAF* mutations had the best survival outcomes among all *BRAF* subgroups. Our findings suggested the potential differential treatment strategies for patients with different *BRAF* mutation subtypes and emphasized the urgency of the development of anti-BRAF drugs, especially targeting the active dimeric BRAF proteins.

## Supplementary Information

**Additional file 1:** Supplementary Methods.** Supplementary Table S1.** Clinical characteristics of patients. **Supplementary Figure S1**. The overview of patients enrolled in this study and the concurrent gene/pathway alterations. (A) A total of 328 colorectal patients whose treatment-naïve tumor samples harboring *BRAF *mutations from the database are included in this study. (B) The oncoprint of the most frequently concurrent gene mutations (top panel) and pathway alterations (bottom panel) are shown as legend by *BRAF* classes. **Supplementary Figure S2. **Patients with Class 3 *BRAF *mutations demonstrated longer OS compared to those with Class1/2 *BRAF *mutations. Patients’ overall survival (OS) data from an additional external cohort were analyzed based on the Kaplan-Meier modeling. NR, not reached.

## Data Availability

All data generated or analyzed during this study are included in this published article and its supplementary information files.

## Abbreviations

CRC: Colorectal cancer
NGS: Next-generation sequencing
AF: Allele frequency
TMB: Tumor mutation burden
MSI-H: Microsatellite instability-high
PFS: Progression-free survival
OS: Overall survival
NER: Nucleotide excision repair
APOBEC: Apolipoprotein B mRNA editing catalytic polypeptide-like enzyme
