# Supplementary material for: The clinical and genomic distinctions of Class1/2/3 BRAF-mutant colorectal cancer and differential prognoses
Source: Biomark Res. 2023 Jan 25;11:11. doi: 10.1186/s40364-022-00443-8 (PMC9875443; doi:10.1186/s40364-022-00443-8)
Supplement: Supplementary file 1 — Additional file 1: Supplementary Methods. Supplementary Table S1. Clinical characteristics of patients. Supplementary Figure S1. The overview of patients enrolled in this study and the concurrent gene/pathway alterations. (A) A total of 328 colorectal patients whose treatment-naïve tumor samples harboring BRAF mutations from the database are included in this study. (B) The oncoprint of the most frequently concurrent gene mutations (top panel) and pathway alterations (bottom panel) are shown as legend by BRAF classes. Supplementary Figure S2. Patients with Class 3 BRAF mutations demonstrated longer OS compared to those with Class1/2 BRAF mutations. Patients’ overall survival (OS) data from an additional external cohort were analyzed based on the Kaplan-Meier modeling. NR, not reached. [file 40364_2022_443_MOESM1_ESM.docx]

**Supplementary File**

**Supplementary Methods**

**Supplementary Table S1.** Clinical characteristics of patients

**Supplementary Figure S1.** The overview of patients enrolled in this study and the concurrent gene/pathway alterations.

**Supplementary Figure S2.** Patients with Class 3 *BRAF* mutations demonstrated longer OS compared to those with Class1/2 *BRAF* mutations

**Supplementary Methods**

*NGS*

A total of 328 treatment-naïve tumor samples with *BRAF* mutations collected from 328 colorectal patients (one sample per patient) were analyzed between June 2015 and June 2020. The comprehensive genomic profiling targeting 425 cancer-relevant genes were conducted by a Clinical Laboratory Improvement Amendments-certified, College of American Pathologists-accredited laboratory (Nanjing Geneseeq Technology, Jiangsu, China) as previously described.(1) In brief, genomic DNA was purified from FFPE samples using the QIAamp DNA FFPE Tissue Kit (Qiagen) and quantified using the dsDNA HS Assay Kit on a Qubit Fluorometer (Life Technologies). Sequencing libraries were prepared using the KAPA Hyper Prep Kit (KAPA Biosystems. Indexed DNA libraries were pooled together for probe-based hybridization capture of the targeted gene regions.

Sequencing was performed using the Illumina HiSeq4000 platform, followed by data analysis as previously described.(2) In brief, low-quality (quality <15) or N bases were removed and then the sequencing reads were mapped to the human reference genome, hg19, using the Burrows-Wheeler Aligner (https://github.com/lh3/bwa/tree/master/bwakit). PCR duplicates were removed by Picard (available at: https://broadinstitute.github.io/picard/). The Genome Analysis Toolkit (GATK) (https://software.broadinstitute.org/gatk/) was used to perform local realignments around indels and base quality reassurance. Common SNPs were excluded if they were present in >1% population frequency in the 1000 Genomes Project or the Exome Aggregation Consortium (ExAC) 65,000 exomes database. The resulting mutation list was further filtered by an in-house list of recurrent artifacts based on a normal pool of whole blood samples and an allele frequency cut-off of 0.5% was applied.

*External public dataset*

A public dataset with survival outcomes of a cohort of unresectable metastatic colorectal patients was downloaded from cBioPortal (<https://www.cbioportal.org/study/summary?id=crc_apc_impact_2020>) on January 24, 2022.(3) Only one tumor sample per patient was included for analysis.

*Statistical analysis and survival analysis*

Data were analyzed using R 3.6.3. Categorical variables between groups were compared using χ2 or Fisher’s exact test. Non-parametric comparisons of TMB and mutational signature were performed using the Mann-Whitney test. Kaplan-Meier method was used to determine median PFS/OS and the significance of survival analysis was determined by the log-rank test.

**Reference**

1. Shu Y, Wu X, Tong X, Wang X, Chang Z, Mao Y, et al. Circulating Tumor DNA Mutation Profiling by Targeted Next Generation Sequencing Provides Guidance for Personalized Treatments in Multiple Cancer Types. Sci Rep. 2017;7(1):583.

2. Yang Z, Yang N, Ou Q, Xiang Y, Jiang T, Wu X, et al. Investigating Novel Resistance Mechanisms to Third-Generation EGFR Tyrosine Kinase Inhibitor Osimertinib in Non-Small Cell Lung Cancer Patients. Clin Cancer Res. 2018;24(13):3097-107.

3. Mondaca S, Walch H, Nandakumar S, Chatila WK, Schultz N, Yaeger R. Specific Mutations in APC, but Not Alterations in DNA Damage Response, Associate With Outcomes of Patients With Metastatic Colorectal Cancer. Gastroenterology. 2020;159(5):1975-8 e4.

**Supplementary Table S1. Clinical characteristics of patients**

|  | **Class 1** | **Class 2** | **Class 3** | ***p***^a^ | ***p***^b^ | ***p***^c^ |
| --- | --- | --- | --- | --- | --- | --- |
| No. of patients | 246 | 29 | 53 |  |  |  |
| Age |  |  |  | 0.550 | 0.155 | 0.135 |
| Median (Range) | 58 (23-93) | 58 (30-72) | 61 (24-90) |  |  |  |
| Gender |  |  |  | 0.320 | 0.355 | 1.000 |
| Male (%) | 143 (58) | 20 (69) | 35 (66) |  |  |  |
| Female (%) | 103 (42) | 9 (31) | 18 (34) |  |  |  |
| Stage at diagnosis |  |  |  | 1.000 | 0.422 | 0.738 |
| I - II (%) | 5 (2) | 0 (0) | 1 (2) |  |  |  |
| III (%) | 25 (10) | 2 (7) | 2 (4) |  |  |  |
| IV (%) | 83 (34) | 9 (31) | 18 (34) |  |  |  |
| Unknown | 133 (54) | 18 (62) | 32 (60) |  |  |  |
| Location |  |  |  | 0.291 | 0.027 | 0.927 |
| Right colon (%) | 60 (24) | 6 (21) | 10 (19) |  |  |  |
| Left colon (%) | 41 (17) | 3 (11) | 7 (13) |  |  |  |
| Rectum (%) | 51 (21) | 10 (34) | 23 (43) |  |  |  |
| Unknown | 94 (38) | 10 (34) | 13 (25) |  |  |  |

Note: ^a^ Class 1 vs. Class 2; ^b^ Class 1 vs. Class 3; ^c^ Class 2 vs. Class 3

**A**

**
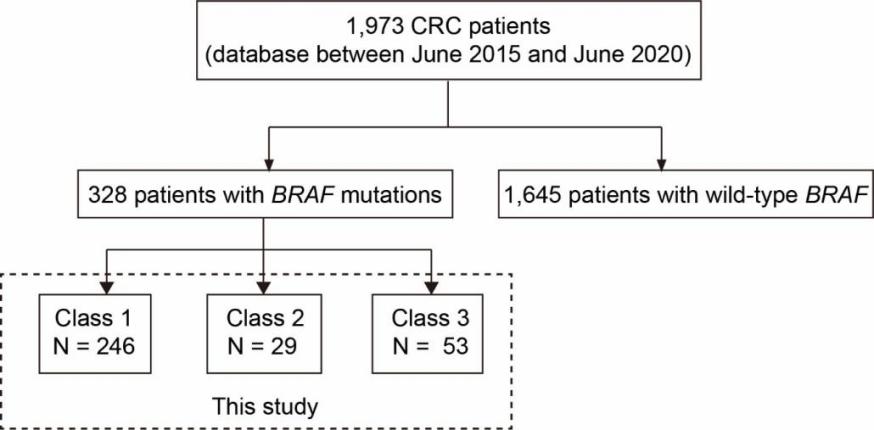
**

**B**


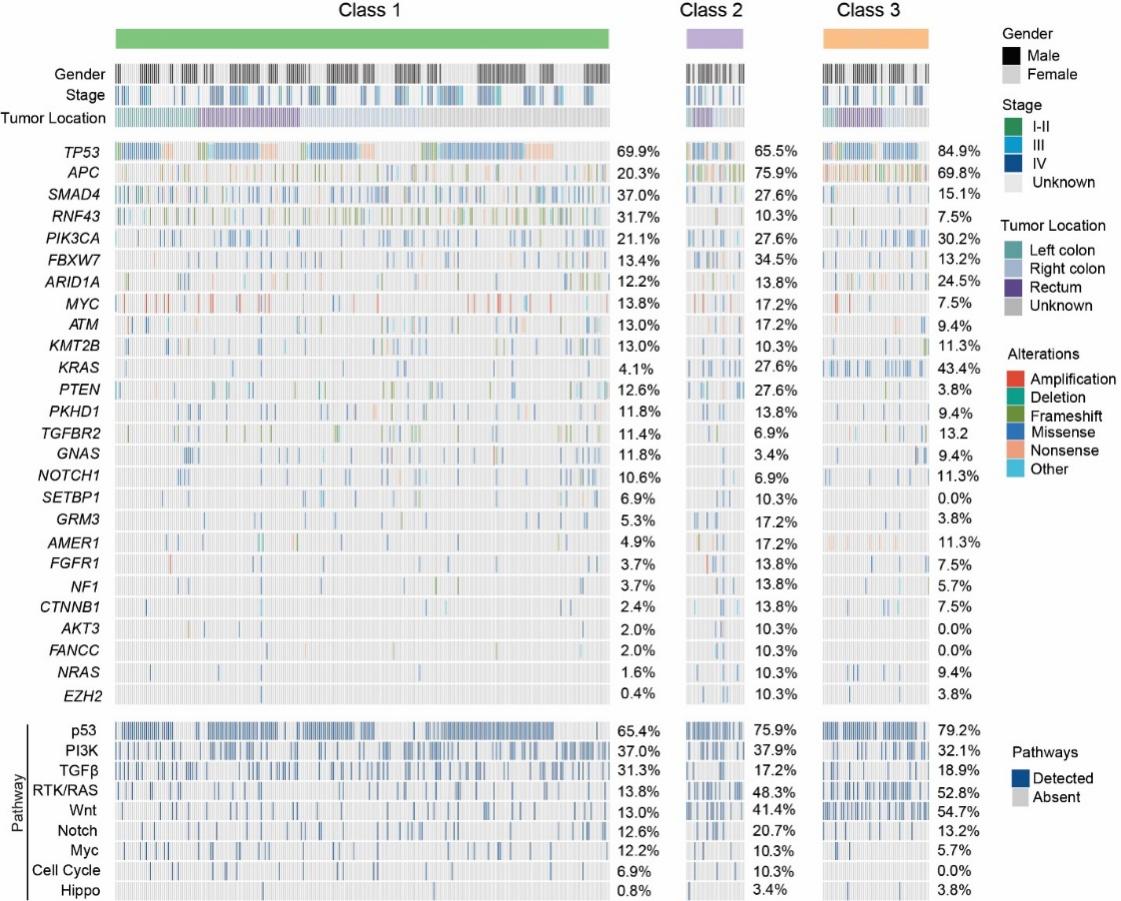


**Supplementary Figure S1. The overview of patients enrolled in this study and the concurrent gene/pathway alterations.**

1. A total of 328 colorectal patients whose treatment-naïve tumor samples harboring *BRAF* mutations from the database are included in this study.
2. The oncoprint of the most frequently concurrent gene mutations (top panel) and pathway alterations (bottom panel) are shown as legend by *BRAF* classes.


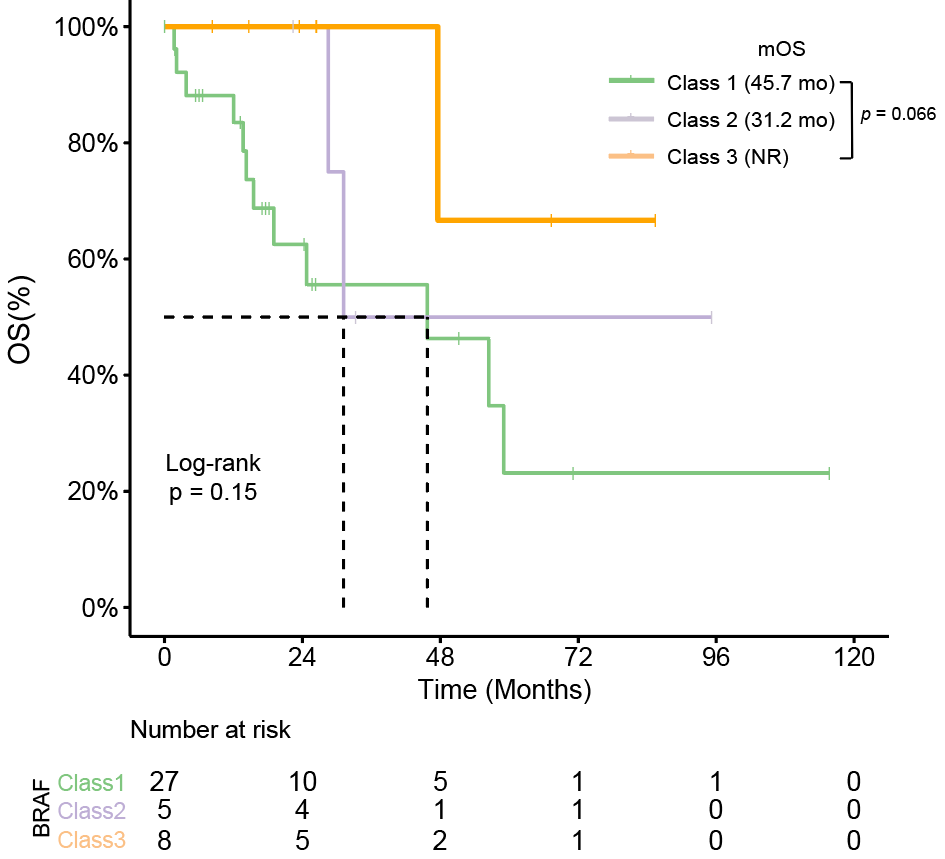


**Supplementary Figure S2. Patients with Class 3 *BRAF* mutations demonstrated longer OS compared to those with Class1/2 *BRAF* mutations.**

Patients’ overall survival (OS) data from an additional external cohort were analyzed based on the Kaplan-Meier modeling. NR, not reached.
